# Supercritical-Carbon Dioxide Fluid Extract from *Chrysanthemum indicum* Enhances Anti-Tumor Effect and Reduces Toxicity of Bleomycin in Tumor-Bearing Mice

**DOI:** 10.3390/ijms18030465

**Published:** 2017-02-24

**Authors:** Hong-Mei Yang, Chao-Yue Sun, Jia-Li Liang, Lie-Qiang Xu, Zhen-Biao Zhang, Dan-Dan Luo, Han-Bin Chen, Yong-Zhong Huang, Qi Wang, David Yue-Wei Lee, Jie Yuan, Yu-Cui Li

**Affiliations:** 1 Guangdong Provincial Key Laboratory of New Drug Development and Research of Chinese Medicine, Guangzhou University of Chinese Medicine, Guangzhou 510006, China; yanghongmei326@gmail.com (H.-M.Y.); yuhongl0822@gmail.com (C.-Y.S.); xing623853793@gmail.com (J.-L.L.); xulieqiang123@gmail.com (L.-Q.X.); Lauyh0822@gmail.com (Z.-B.Z.); zhuojiany38193@gmail.com (D.-D.L.); chenhanbin999@gmail.com (H.-B.C.); jakin3305@gmail.com (Y.-Z.H.); 2 Guangdong New South Artepharm, Co., Ltd., Guangzhou 510006, China; l1393318376@gmail.com; 3 Department of McLean Hospital, Harvard Medical School, Belmont, CA 02478-9106, USA; ywlee228@gmail.com; 4 Dongguan Mathematical Engineering Academy of Chinese Medicine, Guangzhou University of Chinese Medicine, Dongguan 523000, China

**Keywords:** supercritical-carbon dioxide fluid of *C. indicum* (CI_SCFE_), BLM, anti-tumor effect, pulmonary fibrosis, synergism effect

## Abstract

Bleomycin (BLM), a family of anti-tumor drugs, was reported to exhibit severe side effects limiting its usage in clinical treatment. Therefore, finding adjuvants that enhance the anti-tumor effect and reduce the detrimental effect of BLM is a prerequisite. *Chrysanthemum indicum*, an edible flower, possesses abundant bioactivities; the supercritical-carbon dioxide fluid extract from flowers and buds of *C. indicum* (CI_SCFE_) have strong anti-inflammatory, anti-oxidant, and lung protective effects. However, the role of CI_SCFE_ combined with BLM treatment on tumor-bearing mice remains unclear. The present study aimed to investigate the potential synergistic effect and the underlying mechanism of CI_SCFE_ combined with BLM in the treatment of hepatoma 22 (H22) tumor-bearing mice. The results suggested that the oral administration of CI_SCFE_ combined with BLM could markedly prolong the life span, attenuate the BLM-induced pulmonary fibrosis, suppress the production of pro-inflammatory cytokines (interleukin-6), tumor necrosis factor-α, activities of myeloperoxidase, and malondiadehyde. Moreover, CI_SCFE_ combined with BLM promoted the ascites cell apoptosis, the activities of caspases 3 and 8, and up-regulated the protein expression of p53 and down-regulated the transforming growth factor-β1 by activating the gene expression of miR-29b. Taken together, these results indicated that CI_SCFE_ could enhance the anti-cancer activity of BLM and reduce the BLM-induced pulmonary injury in H22 tumor-bearing mice, rendering it as a potential adjuvant drug with chemotherapy after further investigation in the future.

## 1. Introduction

Bleomycin (BLM), a glycopetide originally isolated from *Streptomyces verticillus* [1], is a clinical anti-cancer drug primarily used for the treatment of hepatocellular carcinoma (HCC) and nasopharyngeal carcinoma (NPC). The anti-tumor mechanism mainly consists of inducing DNA damage and has been demonstrated to be mediated through the induction of oxidative stress [1]. Several studies revealed that BLM is vital in the clinical treatment of HCC. However, BLM exhibits the main side effect of dose-dependent pulmonary toxicity, which affects 20% of treated individuals. Pulmonary fibrosis is a severe form of lung toxicity, which was induced by BLM [2]. However, the etiology and mechanism of pulmonary fibrosis have not yet been elucidated. A number of studies have reported that the combination therapy can not only enhance the anticancer effect, but also attenuate the toxicity side-effects to the organs [3,4]. Therefore, the development of a drug that confers lung protection during BLM treatment and improves the chemotherapeutic efficacy of BLM in cancer is essential.

The integration of different signaling pathways plays a critical role in the normal development and tissue homeostasis of metazoans. When one or more signals fail to integrate, the entire signaling network might collapse resulting in diseases, especially cancer [5]. The loss of cross-talk among the two most critical pathways—tumor suppressor Trp53 (p53) and tumor growth factor beta (TGF-β) signaling leads to many kinds of tumors and organ fibrosis [6,7]. p53 can suppress the TGF-β signal, thereby inhibiting the microRNAs (miRNAs) such as miR-17-92/miR-106b-25 clusters to retain the integrity of the antitumor signals. Loss of p53 can lead to the loss of TGF-β receptor 2 (TGFBR2) and miR-34a expression, resulting in attenuated antiproliferative signals [8]. Sun et al. reported that p53 was essential for doxorubicin-induced apoptosis via the TGF-β signaling pathway in osteosarcoma-derived cells [9]. On the other hand, p53 was required for the expression of plasminogen activator inhibitor-1 (PAI-1), a major TGF-β1 target gene and a key causative element in fibrotic disorders [10]. Moreover, Wang et al. found that astaxanthin ameliorated lung fibrosis in rat by regulating the cross-talk between p53 and TGF-β signaling [11]. Thus, substances with an effect on the regulation of p53 and TGF-β signaling pathways may be beneficial for improving the chemotherapeutic efficacy of BLM or alleviating the pulmonary toxicity induced by BLM.

*Chrysanthemum indicum* (*C. indicum*) Linné, a traditional medicinal and edible flower, is widely used as herbal tea, alcoholic beverage, and food additive or directly used to treat several infectious diseases and ailments, such as headache, eye diseases, and various immune-related disorders with high efficacy and low toxicity [12,13,14]. Moreover, the essential oil from the flowers possesses anti-bacterial and anti-cancer properties [15]. Importantly, the supercritical-carbon dioxide fluid extract from flowers and buds of *C. indicum* (CI_SCFE_) have been extensively applied not only in many classical prescription, but also used in daily life as functional foods, cosmetics, and beverages [16]. Pongjit et al. reported that CI_SCFE_ has a strong ability to protect against the chemotherapy-induced renal cell damage [17]. In addition, our previous study demonstrated that CI_SCFE_ has a protective effect against lipopolysaccharide-induced lung injury and UV-induced skin injury [18,19]. However, the effective antitumor activity of CI_SCFE_ combined with BLM in vivo remains unclear.

In the present study, we used the classical H22 ascites tumor-bearing mice model [20,21] to explore the potential synergistic effect of CI_SCFE_ combined with BLM and investigate the underlying mechanism in the treatment of cancer.

## 2. Results

### 2.1. Anti-Tumor Activities of CI_SCFE_, BLM, and Their Combination on H22 Tumor-Bearing Mice

**Figure 1:**
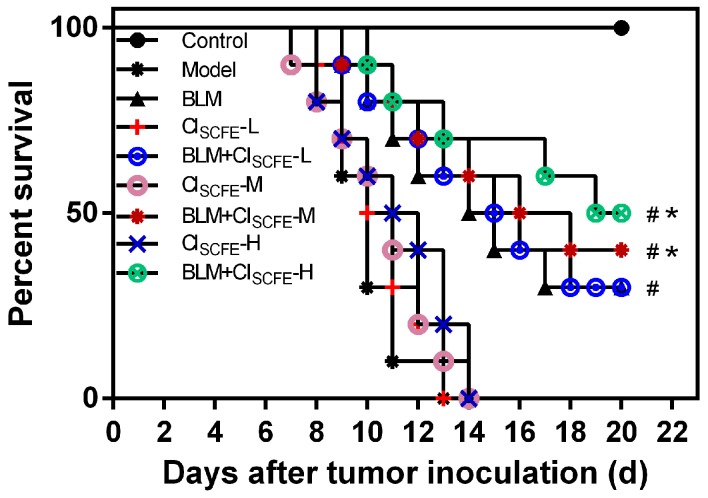
Survivals curve of CI_SCFE_, bleomycin (BLM), and their combination on tumor-bearing mice. The survival rate was followed-up until 22 days after inoculation. Each group comprised of eight mice. ^#^ *p* < 0.05 vs. model group; * *p* < 0.05 vs. BLM group.

To better understand the anti-tumor activities of CI_SCFE_, BLM, and their combination, we evaluated the life span of the H22 tumor-bearing mice model. As shown in Figure 1, compared with the model group, CI_SCFE_ (L: 240 mg/kg, M: 360 mg/kg, H: 480 mg/kg) alone groups exhibited no significant influence on the life-span of the tumor-bearing mice (*p* > 0.05), the BLM alone group could prolong the survival time (*p* < 0.05), while the mice treated with BLM + CI_SCFE_ (M: 360 mg/kg, H: 480 mg/kg) for seven days could significantly prolong the life span as compared to BLM alone (*p* < 0.05). These data suggested that CI_SCFE_-M, H doses could improve the BLM anti-tumor effect. Thus, CI_SCFE_ at a middle dose of 360 mg/kg was used in the subsequent studies.

### 2.2. Synergistic Effect of CI_SCFE_ Combined with BLM on Tumor Growth

**Figure 2:**
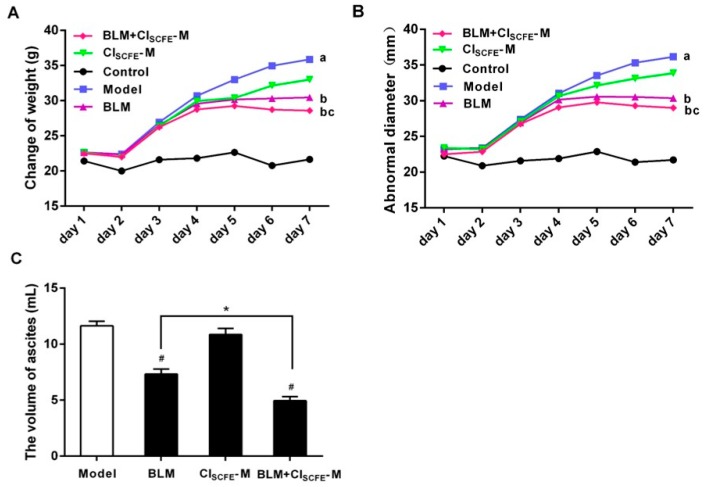
Effect of CI_SCFE_-M, BLM, and their combination on the change of weight (**A**); abnormal diameter (**B**); and ascites (**C**) of tumor-bearing mice. Data represent mean ± SEM (*n* = 10). ^#^ *p* < 0.05 vs. model group; * *p* < 0.05 vs. BLM group. a < 0.05 vs. control group; b < 0.05 vs. model group, c < 0.05 vs. BLM group.

Figure 2 summarizes the effect of CI_SCFE_ combined with BLM on tumor growth. The weight, abnormal diameter, as well as ascites of mice in the model group increased rapidly compared to the control group. On the contrary, the weights, abnormal diameters, and ascites of mice significantly decreased in the BLM alone and BLM + CI_SCFE_-M groups (M: 360 mg/kg), compared to the model group during seven days (*p* < 0.05). The mice in the treated groups showed greater vitality and were in good order, while the combination was more effective than individual treatment. Additionally, no obvious differences were observed in body weight and abnormal diameter between CI_SCFE_-M alone group and model group (*p* > 0.05). These results demonstrated that CI_SCFE_ had little or no effect on tumor-bearing mice; however, it significantly enhanced the anti-tumor activity of BLM.

### 2.3. Synergistic Effect of BLM with CI_SCFE_ in Inducing H22 Ascites Cell Apoptosis

**Figure 3:**
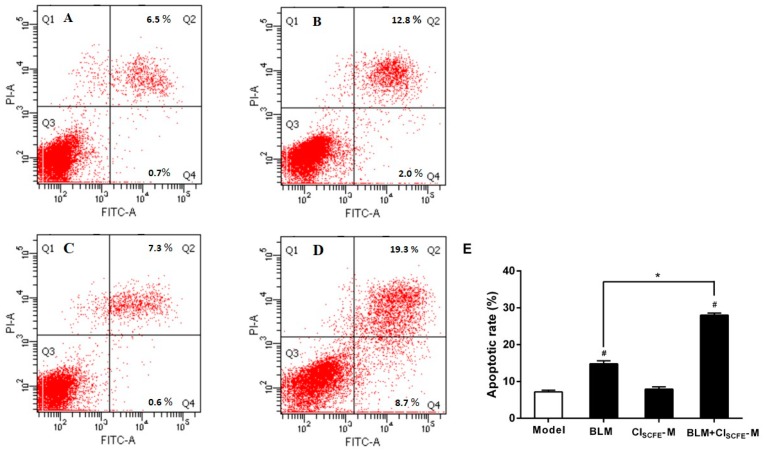
The synergistic effect of BLM with CI_SCFE_ on H22 ascites cell apoptosis induction was analyzed by flow cytometry. (**A**) model group; (**B**) BLM group; (**C**) CI_SCFE_-M group; (**D**) BLM + CI_SCFE_-M group; (**E**) apoptotic rate. The cell populations of Annexin V+/PI− and Annexin V+/PI+ were estimated to represent the total number of apoptotic cells. Data are expressed as mean ± SEM (*n* = 3). ^#^ *p* < 0.05 vs. model group; * *p* < 0.05 vs. BLM group.

To evaluate whether the combination of CI_SCFE_ and BLM can potentially enhance the efficacy of BLM on H22 ascites cell apoptosis, flow cytometry was utilized to assess the rate of apoptosis. As shown in Figure 3, in the BLM treatment alone and BLM + CI_SCFE_-M combined group, the rate of apoptotic cells (Annexin V+/PI− + Annexin V+/PI+) was notably increased as compared to the model group (all *p* < 0.05); the rate of apoptotic cells was obviously increased in the BLM + CI_SCFE_-M combined group as compared to BLM alone (*p* < 0.05). All results suggested that CI_SCFE_ combined with BLM could remarkably increase the H22 ascites cell apoptosis.

### 2.4. CI_SCFE_ Enhanced the Anti-Tumor Effect of BLM by Modulating the Activities of Caspase 3 and Caspase 8

**Figure 4:**
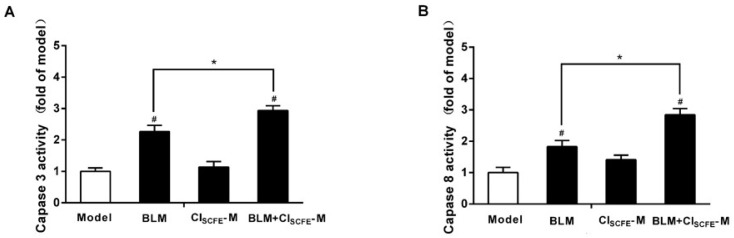
CI_SCFE_ enhanced the anti-tumor effect of BLM by modulating the activities of caspase 3 and caspase 8. The activities of apoptotic performer caspase 3 (**A**); and caspase 8 (**B**) were measured. Data are presented as mean ± SEM of the changes compared to the model group (*n* = 8). ^#^ *p* < 0.05 vs. model group; * *p* < 0.05 vs. BLM group.

Previous studies have demonstrated that caspase families play a vital role in tumor cell apoptosis, including caspase 3 and caspase 8. Figure 4 shows that the activities of caspase 3 (A) and caspase 8 (B) were significantly up-regulated in the BLM alone group and the BLM + CI_SCFE_-M combined group as compared to the model group (all *p* < 0.05). Furthermore, the combination of BLM + CI_SCFE_-M had a statistically stronger effect than BLM alone (all *p* < 0.05). These results suggested that CI_SCFE_ combined with BLM enhanced the effect of BLM on caspase 3 and caspase 8 activities.

### 2.5. Effect of CI_SCFE_ Attenuated BLM-Induced Lung Fibrosis

**Figure 5:**
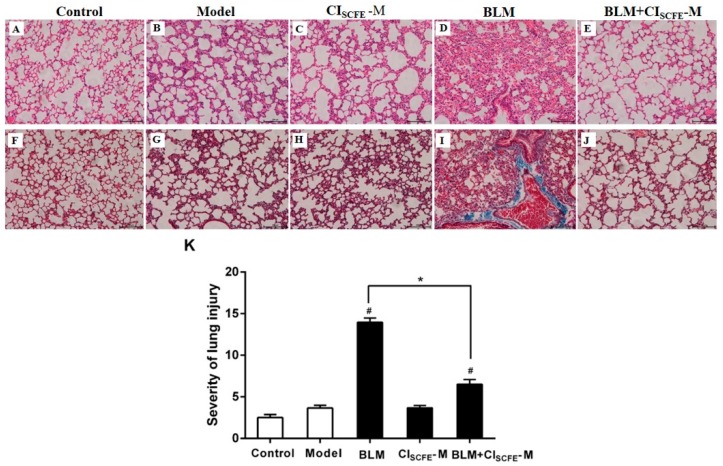
Effect of CI_SCFE_ attenuated BLM-induced lung fibrosis. Lung tissue sections were stained with haematoxylin-eosin (H&E) (**A**–**E**) for pathological examination (200×); Masson (**F**–**J**) for collagen deposition (200×); (**K**) severity scores of lung injury. The slides were histopathologically evaluated using a semiquantitative scoring method. The total lung injury score was estimated by adding up the individual scores of each category. Scale bar indicates 100 μm. Data are expressed as mean ± SEM (*n* = 8). ^#^ *p* < 0.05 vs. model group; * *p* < 0.05 vs. BLM group.

As shown in Figure 5, the lung tissues presented normal structure with no inflammatory, pathological, or collagen deposition in the control group (Figure 5A,F). In comparison with the control group, the model group (Figure 5B,G) and the CI_SCFE_ alone group (Figure 5C,H) showed no obvious pathological changes, whereas in the BLM alone group (Figure 5D,I), haematoxylin-eosin (H&E) staining presented obvious pulmonary injury, including alveolar wall, alveolar, vascular congestion, and inflammatory cell infiltration. Moreover, Masson’s trichrome staining suggested that BLM alone group had massive collagen deposition in the lung interstitium and around the bronchioles as compared to the model group. On the other hand, the combination of BLM + CI_SCFE_-M had remarkably attenuated the pulmonary inflammatory damage and fibrosis as compared to the BLM alone group (Figure 5E,J). In addition, the severity of lung injury was analyzed by H&E staining. As shown in Figure 5K, the model group displayed no obvious pulmonary injury as compared to the control group (*p* > 0.05).

### 2.6. Effect of CI_SCFE_ on Cytokine Production Induced by BLM in the Lung Tissues

**Figure 6:**
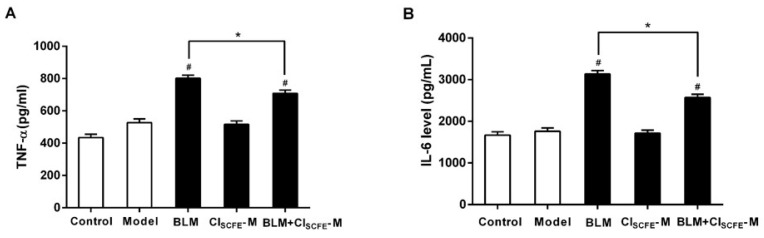
Effect of CI_SCFE_ on BLM-induced cytokines productions in lung tissues. The activities of tumor necrosis factor-alpha (TNF-α) (**A**); and interleukin (IL-6) (**B**) in lungs are presented as the mean ± SEM (*n* = 8). ^#^ *p* < 0.05 vs. model group; * *p* < 0.05 vs. BLM group.

To evaluate the extent of inflammation in lung tissues, the productions of tumor necrosis factor-alpha (TNF-α) and interleukin (IL-6) were measured. Figure 6 demonstrated a remarkable increase in the levels of TNF-α (A) and IL-6 (B) in the BLM-induced lung injuries as compared to the model group (*p <* 0.05, respectively). Conversely, the combination group dramatically decreased the production of these cytokines as compared to the BLM alone group (*p* < 0.05, respectively). No significant differences were observed between the model and CI_SCFE_ alone groups.

### 2.7. Effect of CI_SCFE_ on BLM-Induced Oxidative Stress

**Figure 7:**
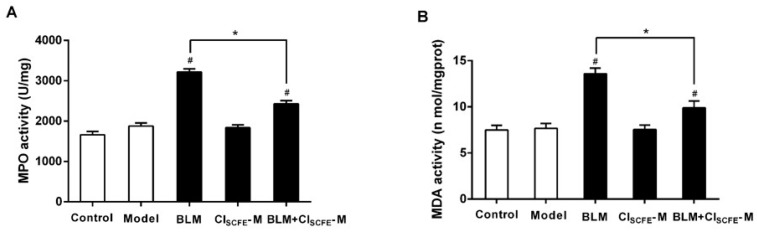
Effect of CI_SCFE_ on BLM-induced oxidative stress. (**A**) Myeloperoxidase (MPO) activity; (**B**) malondialdehyde (MDA) activity. Data are represented as mean ± SEM (*n* = 8). ^#^ *p* < 0.05 vs. model group; * *p* < 0.05 vs. BLM group.

To explore whether the protection effect of CI_SCFE_ against BLM-induced lung injury was related to anti-oxidative effect, the activities of myeloperoxidase (MPO) and malondialdehyde (MDA) were assayed. As shown in Figure 7, treatment with BLM alone notably increased the levels of MPO (A) and MDA (B) as compared to the model group (*p <* 0.05, respectively). However, coupling BLM with CI_SCFE_ remarkably decreased the MPO and MDA activities when compared with BLM alone (*p* < 0.05, respectively), indicating that CI_SCFE_ could decrease the BLM-induced oxidative stress.

### 2.8. Effect of CI_SCFE_ and BLM Treatments on p53 and TGF-β1 Expressions

**Figure 8:**
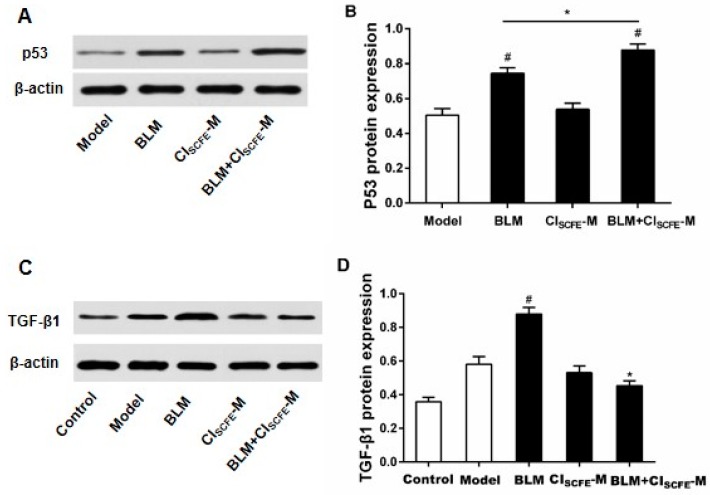
Effect of CI_SCFE_ and BLM treatment on protein-related expression in H22 tumor-bearing mice. The protein expressions of p53 (**A**) and tumor growth factor-beta (TGF-β)1 (**C**) were determined by Western blot. Data (**B**,**D**) are presented as mean ± SEM (*n* = 4). ^#^ *p* < 0.05 vs. model group; * *p* < 0.05 vs. BLM group.

As shown in Figure 8, when compared with the model group, the protein expression of p53 (A) in the ascites cells of BLM-treated mice was apparently up-regulated (*p* < 0.05), whereas the combination of BLM with CI_SCFE_ significantly increased the p53 expression as compared to the BLM alone group (*p* < 0.05). On the other hand, the combination of BLM with CI_SCFE_ could significantly inhibit the TGF-β1 (B) expression in lungs when compared with the BLM alone group (*p* < 0.05).

### 2.9. Expression of miR-29b in Ascites Cells and Lung Tissues

**Figure 9:**
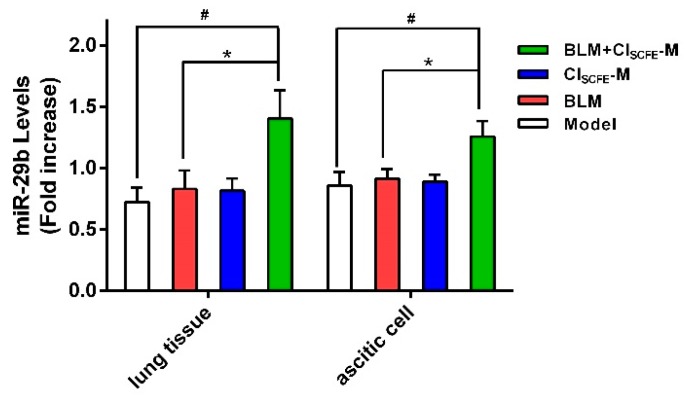
miR-29b expression in lung tissues and ascites cells of H22 tumor-bearing mice. Data are mean ± SEM (*n* = 4). ^#^ *p* < 0.05 vs. model group; * *p* < 0.05 vs. BLM group.

miR-29b is a well-established vital tumor suppressor and fibrosis modulator [22,23], playing a key role in cancer with visceral fibrosis. Thus, we attempted to evaluate whether CI_SCFE_ could modulate the miR-29b expression of BLM-treated tumor-bearing mice. As shown in Figure 9, the treatment with CI_SCFE_ or BLM alone did not exhibit any distinct effect on miR-29b expression in the ascites cells and lung tissues as compared to the model group (*p* < 0.05, respectively). However, the combination of BLM with CI_SCFE_ significantly enhanced the expression of miR-29b in ascites cells and lung tissues as compared to BLM alone (*p* < 0.05).

## 3. Discussion

The clinical usage of chemotherapeutics is well-known to exert severe side-effects [24]. Therefore, discovering and developing adjunctive agents with physiological activities has become an international topic of intensive medical research [25]. BLM is an established anti-cancer drug mainly used in the treatment of HCC and NPC. It has also been demonstrated to be associated with other cytotoxic reagents for the treatment of testis cancer and Hodgkin disease, and these two diseases have a high cure rate obtained by chemotherapy [26]. Interestingly, it is crucial that the main advantage of BLM is neither immunosuppression nor myelosuppression. Thus, the development of an adjuvant is imperative to not only improve the antitumor effect but also attenuate the side-effects of BLM. *C. indicum*, a traditional medicinal and edible flower, possesses heat clearing and toxin-removal activities. Modern pharmacological research displayed that CI_SCFE_ has anti-bacterial, anti-virus, anti-inflammation, anti-sympathetic, anti-oxidant, and anti-neoplastic functions [27,28]. These data suggested that CI_SCFE_ alone exerted no apparent anti-tumor effect in the mice. However, CI_SCFE_ combined with BLM groups markedly prolonged the life-span and significantly decreased the change of weight, abnormal diameter, as well as ascites fluid compared to the BLM alone group. These results suggested that CI_SCFE_ could be a potential adjuvant for BLM to enhance the anti-cancer effect in tumor-bearing mice.

In order to further understand the mechanism of CI_SCFE_ enhanced anti-cancer activity of BLM, we sought to investigate the tumor cell apoptosis. Apoptosis is involved in many physiological processes, which can exclude the abnormal or damaged cells, promoting cancer cell apoptosis as an efficient method to control the tumor growth [29]. Previous studies have revealed that CI_SCFE_ can induce apoptosis and inhibit cell proliferation through signal transducers and activators of transcription factors-3 (STAT-3) and NF-κB signaling pathways in different cancer cell lines [30,31]. Our results showed that CI_SCFE_ combined with BLM could remarkably increase the H22 ascites cell apoptosis. Caspase 8 as a critical pro-apoptotic molecule, which, once activated, can trigger the downstream caspase cascade, including the major cell apoptosis executor caspase 3 [32], Thus, the caspase 3 with caspase 8 activities were measured in the present study to investigate the underlying mechanism of the effect of CI_SCFE_. Furthermore, p53 is a crucial apoptotic protein that directly mediates the downstream caspase 3 and caspase 8 to exert its effect on cell apoptosis. Furthermore, p53 is also a vital tumor suppressor, inactivated in most human cancers with high mutations [33], and can induce the down-regulation of specific proteins. Here, we found that in the CI_SCFE_ combined with BLM groups, the expression of p53 was notably enhanced as compared to BLM alone, with the markedly increased promotion of apoptosis in cancer cells, accompanied by enhanced activities of caspases 3 and 8. These findings imply that the mechanism of CI_SCFE_ combined with BLM in inducing apoptosis of H22 ascites tumor cells might also involve the activation of the p53 apoptotic pathway.

Based on above findings, CI_SCFE_ alone group had no obvious effect on cells apoptosis; however, when coupled with BLM, CI_SCFE_ remarkably promoted the survival rate of H22 tumor-bearing mice. Could it also be caused by reducing the detrimental effect of BLM? It is well-known that one of the most serious side-effects of BLM is pulmonary fibrosis, which in turn increases the mortality rate of BLM-treated patients [34]. A pulmonary pathological slide of lung tissue revealed an obvious pulmonary fibrosis for BLM treatment alone after seven days, whereas the symptoms alleviated in the slides from the combined group of BLM with CI_SCFE_. Pulmonary fibrosis is a chronic fibrosis interstitial lung disease with poor prognosis and unknown etiology. The pro-inflammatory cytokines are known to play a significant role in the processing of pulmonary fibrosis, including TNF-α and IL-6. Furthermore, the excessive free radicals will result in oxidative stress or chronic inflammation [1,2,35], the related enzymes have a significant function in the processing of lung fibrosis, and thus, the activities of the oxidant enzymes (MPO and MDA) were also measured in this study. The results showed that CI_SCFE_ combined with BLM could distinctly decrease the levels of inflammatory cytokines (TNF-α, IL-6) and oxidant enzymes (MPO, MDA). Moreover, previous data reported that TGF-β1 suppressed the release of pro-inflammatory cytokines including TNF-α, IL-6, and itself. In turn, TNF-α and IL-6 stimulated the activity of the TGF-β1 [36]. Consecutively, TGF-β1 inhibited and adjusted the balance of MDA levels with MPO activity in lung tissues [37]. In the present study, the expression of TGF-β1 was evidently decreased in the CI_SCFE_ combined BLM group as compared to BLM alone, thereby proving that CI_SCFE_ could relieve the side-effects of BLM.

P53 and TGF-β1 signaling pathways are equally important in the initiation of cancer, due to abundant cross-talk [38]. In the tumor progression, TGF-β1 and p53 cooperate to regulate anti-proliferation and apoptotic effects under normal conditions. TGF-β1 signaling pathway exhibited a major role in the early anti-tumor function and late-promoting effects [39]. If p53 harbors mutations, the expression of TGF-β1 will be abnormally enhanced and result in cancer. Previous studies have demonstrated that the balance of p53 and TGF-β1 signaling pathways was modulated by the integration of different factors, including other signaling pathways, proteins, chemokines, and miRNAs, of which the participation of miRNAs’ has become increasingly important in recent studies.

miR-29b, a vital tumor suppressor, can suppress the tumor cell growth by regulating the expression of p53 [40], thereby playing a critical role in the process of fibrosis diseases in various tissues, including liver [41], lung [42], kidney [43], and heart [44]. In the tumor procession, miR-29b could regulate the balance of p53 and TGF-β1 signaling pathways simultaneously. The TGF-β1 signaling pathway could, in turn, modulate the activity of miR-29b [8]. Intriguingly, miR-29b could upregulate the level of p53 [40], and consequently activate downstream the p53 pathway including caspases 3 and 8, which eventually induce the apoptosis of tumor cells [45]. In addition, the development of tumors often accompanies chronic inflammation and oxidant stress [46], and thus, the inflammatory cytokines and oxidant enzymes also play a significant role in tumor development. The normal activities of oxidant enzymes (MPO, MDA) could promote cell proliferation; however, the abnormal expression would upregulate the expression of p53 and miR-29b, inducing cell apoptosis [47]. The inflammatory cytokines also play a pivotal role in the procession and metastasis in the tumor, whereby the excess inflammatory factors would upregulate the miR-29b, p53, and caspases 3 and 8 levels to stimulate the tumor cell apoptosis [47,48,49]. Cui et al. [46] demonstrated that the expression of p53 was upregulated in inflamed tissues, and then, p53 negatively regulated the pro-inflammatory factors. Moreover, the pro-inflammatory factors stimulated the expression of miR-29b and enhanced the anti-tumor effect mediated by enhancing the cell apoptosis [8]. On the other hand, miR-29b suppressed the expression of TGF-β1 to mediate the procession of pulmonary fibrosis [22]. The present data substantiated that the treatment with CI_SCFE_ or BLM alone had no obvious effect on miR-29b expression in the ascites cells and lung tissues. Interestingly, the expression of miR-29b levels was dramatically enhanced when CI_SCFE_ was coupled with BLM. These results indicated that CI_SCFE_ combined with BLM affected the miR-29b expression, and regulated the balance between p53 and TGF-β1 signaling pathways in H22 tumor-bearing mice.

## 4. Experimental Section

### 4.1. Materials

Bleomycin (BLM) hydrochloride was purchased from Haizheng Pharmaceuticals (Zhejiang, China). Mouse tumor necrosis factor-α (TNF-α) and interleukin-6 (IL-6) the enzyme-linked immunosorbent assay (ELISA) reagents were purchased from eBioscience (San Diego, CA, USA); Myeloperoxidase (MPO) and malondiadehyde (MDA) Colorimetric Activity Assay Kits were obtained from Jiancheng Institution of Biotechnology (Nanjing, China). Medium RPMI 1640 and fetal bovine serum (FBS) were purchased from Gibco (Grand Island, NY, USA); Penicillin-Streptomycin were obtained from Hyclone (Logan, UT, USA); The Annexin V-fluorescein isothiocyanate (FITC) apoptosis kit was offered by Keygen Biotech (Nanjing, China); TRIzol reagent was offered by Invitrogen Life Technologies (Shanghai, China). All other chemicals and reagents used in the study were of analytical grade.

### 4.2. Preparation of CI_SCFE_

The supercritical fluid CO_2_ extract of *Chrysanthemum indicum* (CI_SCFE_) was prepared and offered by the Institute of New Drug Research & Development Guangzhou University of Chinese Medicine (Lot. 20121104) [19]*.* According to our previous report, the composition analysis was done by combining Gas Chromatography-Mass Spectrometer (GC-MS) and high-performance liquid chromatography with Photodiode Array Detector (HPLC-PAD). Thirty compounds were detected by GC-MS, four compounds were identified by HPLC-PAD (the brief analysis methods and chemical profile of CI_SCFE_ are presented in the Supplementary Materials). In the present study, the CI_SCFE_ was suspended in 3% Tween 80 and confected into different concentration solution. BLM was dissolved in normal saline.

### 4.3. Cell Culture

The mouse H22 hepatocellular carcinoma cells used in this study were purchased from American Type Culture Collection (Rockville, MD, USA) and were revived at 37 °C, then maintained at Dulbecco’s Modified Eagle’s medium (obtained RPMI-1640 medium supplemented with 10% FBS and 1% Penicillin-Streptomycin) in a humidified atmosphere with 5% CO_2_. The cells were incubated in RPMI1640 medium until they reached approximately 2 × 10^6^ cells/mL and 80% viability.

### 4.4. Animals

Male Kunming (KM) mice (18–22 g) were purchased from the Experimental Animal Center, Institute of Guangzhou University of Chinese Medicine (Certificate number SCXK2008-0020; Ethical permission date was September 21, 2015, Guangzhou, China). The animals were housed in a 12-h light/dark cycle under a constant temperature of 24 °C and relative humidity of 65% ± 15%, and fed with standard diet and tap water. The animal experiments were conducted according to the guidelines established by the National Institutes of Health (NIH) Guide for the Care and Use of Laboratory Animals. The procedures were approved by the Animal Care and Welfare Committee of Guangzhou University of Chinese Medicine.

### 4.5. Animal Experiments

H22 cells (2 × 10^6^ cells/mL) were inoculated through abdomen into male KM mice and the ascites cells were passaged three times in the mice, after seven days, the ascites fluid was extracted and diluted with normal saline; the cell concentration was adjusted to 2 × 10^6^ cells/mL and injected into each animal. After five days, 90 mice were randomly divided into nine groups with 10 mice in each group: the control group (normal saline, intraperitoneal (ip) injection), model group (normal saline, ip), BLM alone group (7.5 mg/kg, ip), CI_SCFE_-L, M, H doses alone group (240, 360, 480 mg/kg, respectively, intragastrical (ig) administration), and BLM (7.5 mg/kg, ip) combined with CI_SCFE_-L, M, H doses group (240, 360, 480 mg/kg, respectively, ig). After 24 h, the control and model were intraperitoneally injected normal saline, and BLM alone group with BLM. The CI_SCFE_ alone group were respectively gavaged CI_SCFE_-L, M, H solvent; the BLM combined with CI_SCFE_ groups were respectively intraperitoneally injected BLM and gavaged CI_SCFE_-L, M, H solvent once per day for a total of seven consecutive days. All the mice were allowed free access to water and food until death, and the survival rate was calculated.

Another 50 mice were randomly divided into five groups with 10 mice in each group: the control and model group (normal saline, ip), BLM alone group (7.5 mg/kg, ip), CI_SCFE_-M doses alone group (360 mg/kg, ig), and BLM (7.5 mg/kg, ip) combined with CI_SCFE_-M dose group (360 mg/kg, ig). After 24 h, the control and model groups were intraperitoneally injected with normal saline and the BLM alone group was intraperitoneally injected with BLM; the CI_SCFE_ alone group was gavaged CI_SCFE_-M solvent; the BLM combined with CI_SCFE_ groups was intraperitoneally injected BLM and gavaged CI_SCFE_-M solvent once per day for a total of seven consecutive days. At day 8, 10 mice of each group were executed and ascites were collected with lung tissues for the subsequent tests. A portion of the ascites was solubilized in the TRIzol reagent for the extraction of total RNA, and the other portion was used for Western blotting analysis. The lung tissue was rapidly removed and washed in ice-cold normal saline, snap-frozen in liquid nitrogen, and stored at −80 °C until further analysis.

### 4.6. Histopathological Examination

Lung tissues were fixed in 10% neutral buffered formalin and embedded in paraffin wax, cut into 5 μm thick slices, and subjected to haematoxylin-eosin (H&E) staining and Masson’s trichrome staining to detect inflammation or collagen deposition, respectively. The lung injuries were ranked from 0 (normal) to 4 (severe) for four categories: congestion, edema, interstitial inflammation, and inflammatory cell infiltration; the overall lung injury score was calculated by adding up the individual scores of each category [50].

### 4.7. Determination of MPO, MDA Activities

Lung tissues (0.2 g) were homogenized (1000 rpm, 30 s) with steel shot in nine volumes of cold normal saline (4 °C), followed by centrifugation at 3000× *g* for 10 min at 4 °C. The total supernatant was extracted, and subsequently, the enzyme-linked immunosorbent assay (ELISA) kits (Jianchen Institution of Biotechnology, Nanjing, China) were utilized for the estimation of activities of MPO and MDA in the lung tissues.

### 4.8. Evaluation of the Expression of TNF-α and IL-6

Other lung tissues (0.3 g) were homogenized (1000 rpm, 30 s) with steel shot in 9 volumes of cold PBS (4 °C) and centrifuged at 3000× *g* for 10 min at 4 °C. The total supernatant was extracted to estimate the secreted TNF-α as well as IL-6 by the operation sequences utilizing ELISA kits.

### 4.9. Flow Cytometry Analysis (FACS)

The ascites fluid was centrifuged (1000 rpm, 3 min) and washed twice using the pre-chilled PBS. The cell concentration was adjusted to 1 × 10^6^ cells/mL PBS. Then, 400 µL Annexin V-FITC and 5 µL Annexin V-FITC were integrated with the cells, respectively, and incubated for 15 min in the dark (4 °C). Subsequently, 10 µL propidium iodide (PI) was lightly mixed with the cells and incubated for 5 min (4 °C), followed by FACS (Becton Dickinson FACS Calibur). The apoptotic cells were quantified as the percentage of sub-G1 DNA content in each sample.

### 4.10. Western Blot Analysis

The ascites cells were washed with cold PBS three times and centrifuged at 1000× *g* for 5 min at 4 °C, the total supernatant was aspirated and 200 µL radio immunoprecipitation assay buffer (RIPA) lysis buffer added and agitated to completely to crack the cells on the ice, followed by centrifugation at 12,000× *g* for 5 min at 4 °C. The total protein was collected in the supernatant. Next, the nuclear and cell plasma protein extraction kits were utilized to isolate the respective protein fractions. The protein concentration was estimated by bicinchoninic acid assay method, an equivalent amount separated on 10% SDS-polyacrylamide gel electrophoresis (PAGE), and transferred to polyvinylidene fluoride (PVDF) membranes. The membranes were blocked for 1 h with PBS containing 5% dried milk powder and incubated overnight at 4 °C with rabbit anti-Mcl-1 (1:100, Santa Cruz Biotechnology, Inc., Shanghai, China), rabbit anti-BAG3 (1:1000), or mouse anti-MMP2 (1:200, DaiichiFine Chemical Co., Ltd., Shanghai, China). Then, the membranes were washed in TBST, and the appropriate horseradish peroxidase (HRP)-conjugated secondary antibodies diluted in TBST were added. The lung tissues and ascites cells were washed two to three times with cold PBS to remove the blood and then homogenized. Approximately, 10 volumes of RIPA lysis buffer was added and mixed vigorously complete cracking of the cells on ice, followed by centrifugation at 12,000× *g* for 5 min at 4 °C; the total protein was collected in the supernatant.

### 4.11. Caspase 3 and Caspase 8 Activities Assay

The activities of caspase 3 and caspase 8 in the H22 ascites cells were evaluated by the respective caspase activity assay kit purchased from Keygen Biotech (Nanjing, China). Both activities were correspondingly assayed by spectrophotometry (λ = 405 nm), according to the manufacture’s protocols.

### 4.12. Real-Time Polymerase Chain Reaction Analysis

H22 ascites fluid, as well as lung tissues, were washed with PBS; the ascites fluid was extracted with TRIzol reagent (Invitrogen, Carlsbad, CA, USA) to isolate the total RNA. Total RNA (1.5 μg) was reverse transcribed using Kit (Applied Biosystems, Branch burg, NJ, USA) to yield cDNA. The reaction was run at 50 °C for 2 min, 95 °C for 10 min, followed by 40 cycles at 95 °C for 15 s and 60 °C for 1 min, the solubility curve 75–95 °C, heat up 1 °C each 20 s on Applied Biosystems Step-One Fast Real-Time PCR system. Glyceraldehyde-3-phosphate dehydrogenase (GAPDH) was used as an internal control. Fold change = 2^−ΔΔ*C*t^, ΔΔ*C*_t_ = (*C*_tSample_ − *C*_tGAPDH_) − (*C*_tControl_ − *C*_tGAPDH_). The primers sequences of gene including miR-29b, GAPDH was synthesized by Invitrogen, the siRNA sequences were listed as follows: 5′-CTCAACTGGTGTCGTGGAGTCGGCAATTCAGTTGAGTCTAAACC-3′; 5′-ACACTCCAGCTGGGGCTGGTTTCATATGGTGG-3′ for miRNA-29b and 5′-CTCGCTTCGGCAGCACA-3′ and 5′-AACGCTTCACGAATTTGCGT-3′ for GAPDH control.

### 4.13. Statistical Analysis

All data were assessed by one-way ANOVA method, Least Significant Difference (LSD), and Dunnett’s T3 (3) test for comparison between any two means; *p* < 0.05 was considered statistically significant. The comparison of survival curves was determined by the log-rank (Mantel-Cox) test. All data were analyzed using the statistical analysis software (SPSS 13.0, New York, NY, USA).

## 5. Conclusions

Our studies confirmed that CI_SCFE_ could enhance the anti-cancer activity of BLM in H22 tumor-bearing mice. The synergistic effect of BLM was related to CI_SCFE_ by inducing apoptosis of H22 ascites tumor cells and reducing the pulmonary injury induced by BLM. Thus, the possible underlying mechanism was associated with the regulation of the balance of p53 and TGF-β1 signaling pathways. These results indicated that CI_SCFE_ could serve as a putative adjuvant drug with chemotherapy in the future.

## Supplementary Materials

Supplementary materials can be found at www.mdpi.com/1422-0067/18/3/465/s1.
