# Supplementary material for: Supercritical-Carbon Dioxide Fluid Extract from Chrysanthemum indicum Enhances Anti-Tumor Effect and Reduces Toxicity of Bleomycin in Tumor-Bearing Mice
Source: Int J Mol Sci. 2017 Feb 24;18(3):465. doi: 10.3390/ijms18030465 (PMC5372490; doi:10.3390/ijms18030465)
Supplement: Supplementary file 1 [file ijms-18-00465-s001.pdf]

Supplementary Materials: Supercritical-Carbon Dioxide Fluid Extract from *Chrysanthemum indicum* Enhances Anti-Tumor Effect and Reduces Toxicity of Bleomycin in Tumor-Bearing Mice

Hong-Mei Yang, Chao-Yue Sun, Jia-Li Liang, Lie-Qiang Xu, Zhen-Biao Zhang, Dan-Dan Luo, Han-Bin Chen, Yong-Zhong Huang, Qi Wang, David Yue-Wei Lee, Jie Yuan and Yu-Cui Li

The methods of gas chromatography-mass spectrometer (GC-MS) and high-performance liquid chromatography with Photodiode Array Detector (HPLC-PAD) analysis for the determination the compositions of CI_{SCFE}

This study aimed to determine the compositions of CI_{SCFE}

1. Materials and methods

1.1. Materials

CI_{SCFE} was offered by the Institute of New Drug Research & Development Guangzhou University of Chinese Medicine (Lot. 20121104). GC-MS was offered by Agilent (Palo Alto, USA), HPLC was purchased from Hewlett Packard (Palo Alto, USA). Quercetin (Lot. 100081-200406), linarin (05-1017), apigenin (713-8712) and acacetin (480-44-4) were provided by Sigma-Aldrich Trading Co., Ltd. (Shanghai, China). Luteolin (130322) was purchased by Biotechnology Co., Ltd. (Chengdu, China), normal hexane, methyl alcohol, phosphoric acid, acetonitrile, and other chemicals were of analytical grade.

1.2. Methods

Along with 3.0 g of CI_{SCFE}, normal hexane (100 mL × 3) and 75% methyl alcohol (100 mL) was added. Next, ultrasound extraction was performed three times and the layer of normal hexane were evaporate concentrated to 20 mL. The portion of normal hexane was analyzed by GC-MS and the was were analyzed by HPLC-PAD.

GC-MS was employed in the Agilent Technologies 7890B GC System, Chromatographic separation was achieved on a 5% phenyl methyl siloxane HP-5MS capillary column (30 cm × 250 μm × 0.25 μm, Agilent). The oven temperature was set initially at 60 °C followed by a gradient of 15 °C/min up to 180 °C (held for 6 min) and then programmed to 220 °C at 10 °C/min (held for 5 min); furthermore, the temperature was up to 260 °C at 15 °C/min (held for 8 min) and finally to 280 °C at 10 °C/min (held for 5 min). No Split injecting samples (1 μL) and helium was used as carrier gas of 1.0 mL/min flow rate. The spectrometer was set in electron impact (EI) mode, the ionization energy was 70 eV, the scan range was 40–400 amu, and the scan rate was 0.34 s per scan. The inlet and ionization source temperatures were 230 and 250 °C, respectively. Identification of the compounds was based on a comparison of retention indices (relative to the retention times of *n*-alkanes on the HP-5MS column) and mass spectra with those of authentic samples, data from, the Wiley/National Bureau of Standards (NBS) Registry of Mass Spectral Data (V.5.0), and the National Institute of Standards and Technology (NIST), and the MS Search (2015, V.2.0). The relative percentage of each compound in the normal hexane layer of CI_{SCFE} was quantified based on the peak area integrated by the analysis program.

HPLC-PAD analysis: HPLC analysis was employed in Agilent1100 HPLC system. The separation was performed on a Kromasil KR100-5 C_{18} column (4.6 mm × 250 mm, 5 μm, Kromasil) with a flow rate of 1.0 mL/min, column temperature at 30°C, and injection volume of 10 μL. The mobile phase consisted of acetonitrile (solvent A), and 0.1% Phosphoric acid (solvent B) was used to elute the targets with the gradient mode (0–5 min: 5%–25% A; 15–25 min: 25%–45% A; 25–28 min: 50%–60% A; 28–33 min: 60%–70% A; 33–40 min: 70%–5% A). Analysis was based on the retention time

and the ultraviolet (UV) absorption (190 to 800 nm). The content of these compounds was quantitatively analyzed with peak areas under the standard curves at 334 nm.

2. Results

2.1 GC-MS Analysis of CI$_{SCFE}$

The results of the GC-MS chromatograph of the normal hexane layers of CI$_{SCFE}$ (shown in Figure S1) clearly verified 30 compositions and all the components are shown in Table S1.

2.2 HPLC Analysis of CI$_{SCFE}$

The results of the HPLC chromatograph of the 75% methyl alcohol layers of CI$_{SCFE}$, shown in Figure S2, clearly verified five compositions which are shown in Table S2.

Table S1. The chemical profile of CI$_{SCFE}$, analyzed by gas chromatography-mass spectrometer (GC-MS) analysis and the relative percentage calculated by integrated peak area in Agilent MSD Chemstation data analysis syster.

Number	Components	Retention Time (R.t)	Percentage (%)
1	Camphor	5.570	0.559
2	alpha-Terpineol	6.033	1.565
3	9-Tetradecenal, (Z)	6.558	0.75
4	Thymol	6.861	0.505
5	*n*-Decanoic acid	7.594	1.181
6	Isoaromadendrene epoxide	9.843	0.784
7	Epiglobulol	10.858	1.011
8	Bornyltiglate	12.213	1.100
9	*n*-Propyl 5,8,11,14,17-eicosapentaenoate	12.917	0.993
10	Phytol	14.294	0.932
11	Hexadecanoic acid	17.236	0.813
12	Pentacosane	18.888	1.950
13	9,12-Octadecadienoic acid (Z,Z)	19.830	0.547
14	Oleic Acid	21.294	0.404
15	Tricosane	23.108	5.271
16	Tetracosane	24.831	0.803
17	Docosanoic acid	25.637	0.448
18	Tetratriacontane	26.189	6.341
19	α-Santonin	27.371	1.053
20	Pentacosane, 13-un20decyl	28.260	0.590
21	P21entacosane	29.027	8.62.3
22	*Z*-5-Methyl-6-heneicosen-11-one	30.426	0.572
23	Triacontane	30.663	1.337
24	Nonacosane	31.940	0.652
25	*E*,*E*,*Z*-1,3,12-Nonadecatriene-5,14-diol	33.212	13.491
26	Heptacosane	35.181	2.885
27	Dotriacontane	36.280	0.937
28	Octacosane	37.163	8.885
29	Octadecane, 3-ethyl-5-(2-ethylbutyl)	37.578	1.769
30	Hexatriacontane	39.055	1.602

Table S2. The chemical profile of CISCFE, the relative percentage calculated by integrated peak area analyzed and quantified by HPLC-PAD analysis system.

Number	Components	Retention Time (R.t)	Percentage (%)
1	Luteolin	16.918	0.725
2	Apigenin	17.313	0.883
3	Querocetin	21.135	0.85
4	Linarin	24.191	0.622
5	Acacetin	33.199	1.05

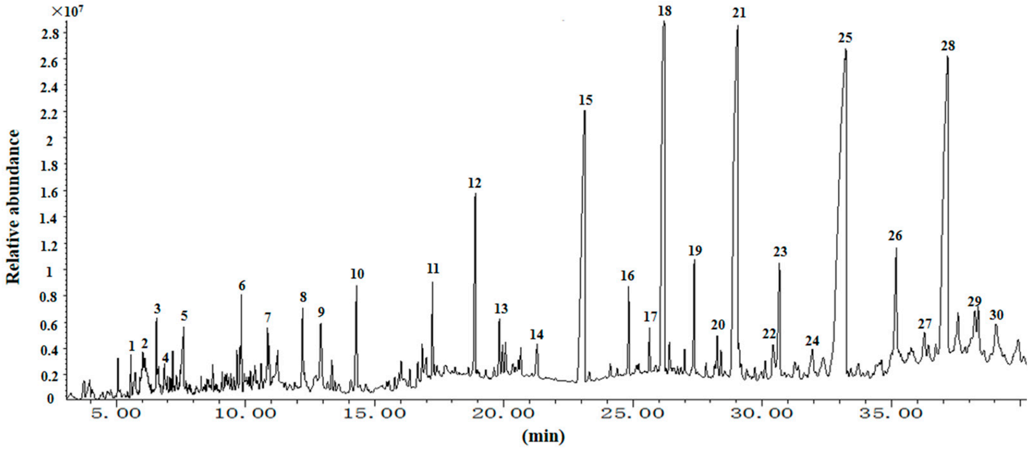

Figure S1. The GC-MS chromatograph of the normal hexane layers of CISCFE.

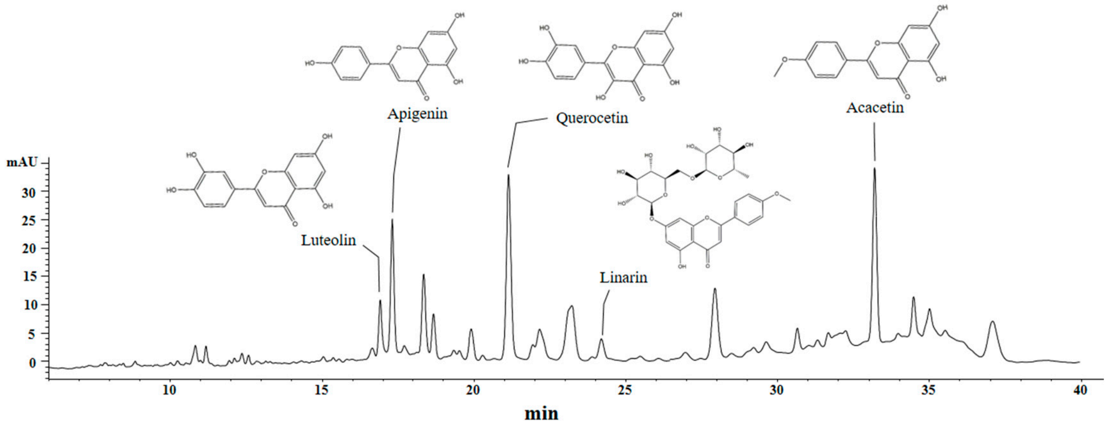

Figure S2. The HPLC chromatograph of the 75% methyl alcohol layers of CISCFE.
